# Modulation of keratin 1, 10 and involucrin expression as part of the complex response of the human keratinocyte cell line HaCaT to ultraviolet radiation

**DOI:** 10.2478/intox-2013-0030

**Published:** 2013-12

**Authors:** Martina Moravcová, Antonín Libra, Jana Dvořáková, Alena Víšková, Tomáš Muthný, Vladimír Velebný, Lukáš Kubala

**Affiliations:** 1 Contipro Biotech s.r.o., Dolní Dobrouč, Czech Republic; 2 Institute of Experimental Biology, Department of Physiology and Immunology of Animals, Faculty of Science, Masaryk University, Brno, Czech Republic; 3 Generi Biotech, Hradec Kralove, Czech Republic; 4 Institute of Biophysics, Academy of Sciences of the Czech Republic, Brno, Czech Republic; 5 International Clinical Research Center – Center of Biomolecular and Cell Engineering, St. Anne's University Hospital Brno, Brno, Czech Republic

**Keywords:** involucrin, keratinocyte, keratin, ultraviolet light, inflammation

## Abstract

Skin exposure to ultraviolet (UV) light evokes a complex stress response in keratinocytes. Keratin filament organization provides structural stability and mechanical integrity of keratinocytes. Involucrin is a transglutaminase substrate protein contributing to the formation of insoluble cornified envelopes. However, a more complex role for keratins and involucrin has been proposed, including the regulation of cell stress response. The aim was to evaluate modulations of keratin 1, 10 and involucrin expression in HaCaT in the light of the complex response of these cells to UV-B radiation, including effects on c-Jun and matrix metalloproteinase 1 (MMP-1) gene expression and production of interleukin (IL) 6 and 8. A UV-B (300±5 nm) dose of 10 mJ/cm^2^ was selected since this dose resulted in a partial decrease in cell viability in contrast to higher UV-B doses, which induced complete cell death 48 h after treatment. The UV-B radiation induced significant expression of keratin 1 and 10 and decreased expression of involucrin. This was accompanied by increased expression of c-Jun and MMP-1 and IL-6 and IL-8 production. The data suggest that the expression of keratin 1, 10 and involucrin is modulated in HaCaT keratinocytes as a part of the complex stress response to UV radiation.

## Introduction

Skin exposure to the UV component of sunlight mediates extensive detrimental effects, including a disrupted inflammatory response, premature aging (photoaging) and cancer (Fisher *et al.*, 1997; Matsumura & Ananthaswamy, 2004; Takashima & Bergstresser, 1996). The acute response of the skin to a high dose of UV light is characterized by the stress response of skin cells, the death of damaged cells, and the induction of inflammatory response (Ichihashi *et al.*, 2003).

The majority of UV-B rays are absorbed in the epidermis and keratinocytes are the most exposed cell type. The complex response of keratinocytes to UV exposure includes increased expression and activation of the transcriptional factor activating protein-1 (AP-1, composed of c-Jun and c-Fos) and nuclear transcriptional factor κB (NF-κB). Among other effects, AP-1 stimulates the production of matrix metalloproteinase (MMPs) in both the epidermis and dermis, leading to degradation of collagen and elastic fibers, which contributes to the photoaging process (Fisher *et al.*, 2002). The activation of NF-κB stimulates the production of pro-inflammatory cytokines, such as tumor necrosis factor-α (TNF-α), interleukin (IL)-1, IL-6, and IL-8. The signaling pathways triggered by these cytokines further stimulate the action of AP-1 and NF-κB and thereby amplify the UV response in keratinocytes, promoting a multifaceted inflammatory response in the skin, involving various cell types (Heck *et al.*, 2004).

There is evidence that the response of keratinocytes to UV light is also associated with changes in the expression of keratins and involucrin. Keratins are the basic material for filaments, whose organization provides structural stability and flexibility and ensures the mechanical integrity of keratinocytes (Coulombe & Lee, 2012; Chamcheu *et al.*, 2012). However, keratins have been suggested to have more complex roles, which are associated with regulatory functions (Coulombe & Lee, 2012; Chamcheu *et al.*, 2012). They form complex signaling networks, interacting with various kinases and adaptor and apoptotic proteins to regulate apoptosis, cell architecture, stress response, protein synthesis, and organelle and vesicle (re)distribution. When keratinocytes begin to stratify and differentiate, they acquire the differentiation-specific keratin 1 (K 1) and keratin 10 (K 10). Interestingly, these keratins are supposed to possess various newly-described functions related to the regulation of cell and tissue growth in the epidermis. *In vitro* experiments indicated a direct involvement of K10 in cell cycle control (Paramio *et al.*, 1999; Paramio *et al.*, 2001). Mutations in either K10 or its partner K1 can contribute to induction of hyperproliferation in the basal layer of the epidermis, hyperplasia in the basal compartment of the epidermis, and hyperkeratosis, as confirmed in K10 deficient mice (Reichelt *et al.*, 2004; Reichelt & Magin, 2002). Furthermore, involucrin, as a protein rich in glutamines and lysines, is important for crosslinking by transglutaminase to build the cornified envelope, which is formed during the terminal maturation of keratinocytes in corneocytes (Kalinin *et al.*, 2002). Both involucrin and keratin expression is modulated in diseases associated with alterations of the terminal differentiation of keratinocytes, *e.g.* psoriasis and cancer (Commandeur *et al.*, 2009; Chen *et al.*, 2013).

Conflicting information is available on the effects of UV radiation on keratinocyte differentiation. Studies of the modulation of keratin and involucrin expression by UV radiation yielded incompatible results (Bernerd & Asselineau, 1997; Bosset *et al.*, 2003; Horio *et al.*, 1993; Lee *et al.*, 2002; Mammone *et al.*, 2000; Smith & Rees, 1994).

The aim of this study was to evaluate the modulation of K 1, K 10 and involucrin expression in the HaCaT human immortalized keratinocyte cell line exposed to UV-B-radiation in the context of the complex response of these cells to UV-B radiation.

## Methods

### Cell cultures, UV-B irradiation, and determination of cell viability

The spontaneously immortalized human keratinocyte cell line HaCaT (gift of Prof. Dr. N. Fusenig (Boukamp *et al.*, 1988)) was grown in Dulbecco's modified Eagle's medium (DMEM, Sigma, St Louis, Missouri) supplemented with 10% fetal bovine serum (BFS, Invitrogen, Carlsbad, CA, USA), glutamine (0.29 mg/ml) (Sigma) and gentamycin (50 µg/ml) (Invitrogen) in 5% CO_2_ at 37 °C, as described previously (Ruszova *et al.*, 2013; Ruszova *et al.*, 2008).

UV-B radiation doses (5 to 50 mJ/cm^2^) were applied as reported previously (Hasova *et al.*), employing a 1000 W xenon solar UV-simulator equipped with a dichroic mirror and a 300±5 nm interference filter (Oriel Instruments, Stratford, CT, USA). Before UV-B treatment, the cell culture medium was replaced by phosphate buffer saline (pH 7.4, PBS). Immediately after UV-B radiation, PBS was replaced by the cell culture medium, and cells and/or cell media were harvested 1, 6, 24 and 48 hours after UV-B radiation. Cell viability was measured using a 3-(4.5-dimethylthiazol-2-yl)-2.5-diphenyl tetrazolium bromide (MTT) assay, as described previously (Vistejnova *et al.*, 2009).

### Determination of gene expression

Total RNA was isolated using Trizol^®^ (Invitrogen) and the synthesis of cDNA was carried out using Revert-Aid H Minus MuLV-Reverse transcriptase (Fermentas, Vilnius, Lithuania) according to the manufacturer's instructions. A quantitative real time reverse transcription polymerase chain reaction (qRT-PCR) was accomplished using Gene Expression TaqMan Assays (c-Jun: Hs_99999141_s1, MMP-1: Hs_00899658_m1, K 1: Hs00196158_m1, K 10: Hs00166289_m1, involucrin: Hs00846307_s1) on employing a Miniopticon RT-PCR system instrument (Bio-Rad, Hercules, CA, USA) at universal cycling conditions (15 minutes at 95 °C, 40 cycles for 15 s at 95 °C, and 1 minute at 60 °C). Cycle threshold values were determined from the correlation factor of the calibration curve data by means of threshold analysis using Bio-Rad Opticon software (Bio-Rad). The threshold cycle (Ct) was determined for genes of interest while actin β was used as a house keeping gene, and the relative amount of mRNA in each sample was calculated based on its Ct value normalized with the Ct value of the housekeeping gene (actin β).

### Determination of cytokine production

The concentration of cytokines in the cell culture supernatant was determined by commercial Enzyme-Linked Immunosorbent Assay kits (Bender MedSystems, Austria) (Hasova *et al.*). The obtained values were related to the amount of viable cells.

### Statistical analysis

The results show the mean ± SEM derived from at least three independent experiments. Analysis was performed with the paired Student‘s t-test, and *p≤*0.05 was considered statistically significant.

## Results

**Figure 1:**
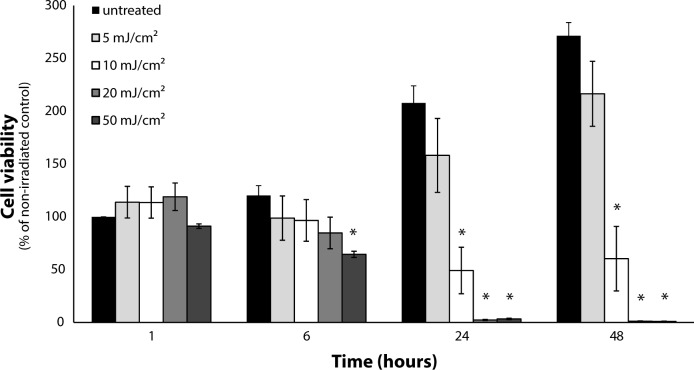
Dose-dependent reduction in human keratinocyte viability induced by UV-B radiation. Keratinocytes were irradiated by UV-B (5, 10, 20, 50 mJ/cm^2^) and cell viability was determined by the MTT assay 1 to 48 hours after UV radiation. Data are expressed as mean ± SEM (n=4). Statistically significant differences (Student's t-test, *p≤*0.05) to the respective non-radiated control group incubated for 1 hour are marked with an asterisk.

The modulation of HaCaT viability by a range of UV-B doses was screened (Figure 1). Doses of 20 and 50 mJ/cm^2^ UV-B radiation induced complete cell death of keratinocytes after 24 hours (Figure 1). A 10 mJ/cm^2^ UV-B dose affected the viability, which however did not fall lower than by approximately 40% even after 48 hours, providing enough surviving cells for further analyses (Figure 1). In contrast, a dose of 5 mJ/cm^2^ did not significantly affect cell numbers compared to non-irradiated control. Thus, the 10 mJ/cm2 dose was selected as a model dose for further evaluation of keratinocyte stress response to UV-B radiation.

**Figure 2:**
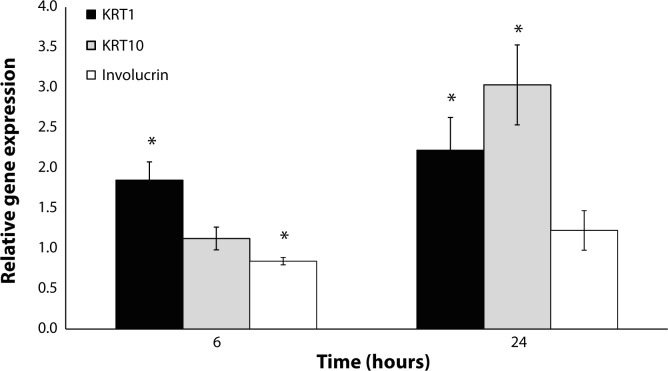
Modulation of the gene expressions of K 1, K10 and involucrin in HaCaT induced 6 and 24 hours after UV-B irradiation (10 mJ/cm^2^). Data are expressed as a percentage of non-irradiated control mean ± SEM (n=6). Statistically significant differences (Student's t-test, *p≤*0.05) to the respective non-radiated control group are marked with an asterisk.

Employing this optimized protocol of the UV-B radiation of HaCaT, the expressions of K 1, K 10 and involucrin were evaluated. Interestingly, UV-B radiation increased the expression of K 1 both 6 and 24 hours after the irradiation (Figure 2). The expression of K 10 was increased significantly only 24 hours after irradiation (Figure 2). In contrast, involucrin expression was significantly decreased 6 hours after irradiation and unchanged after the longer time interval (Figure 2).

**Figure 3:**
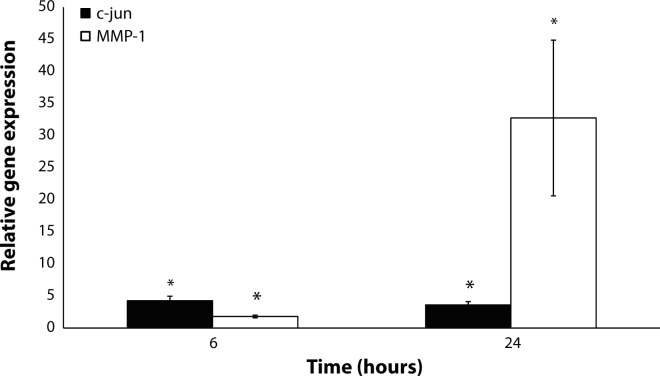
Increases in the gene expressions of c-Jun and MMP-1 in HaCaT induced 6 and 24 hours after UV radiation (10 mJ/cm^2^). Data are expressed as a percentage of non-irradiated control mean ± SEM (n=6). Statistically significant differences (Student's t-test, *p≤*0.05) to the respective non-irradiated control group are marked with an asterisk.

**Figure 4:**
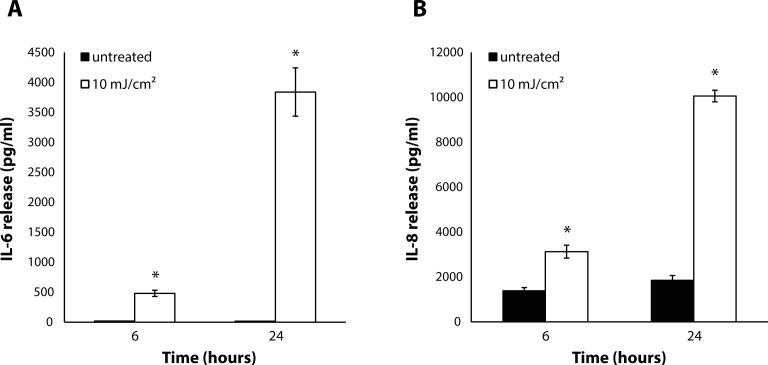
Increases in IL-6 (**A**) and IL-8 (**B**) production by keratinocytes into the medium 6 and 24 after UV radiation (10 mJ/cm^2^). Data are expressed as mean ± SEM (n=3). Statistically significant differences (Student's t-test, *p≤*0.05) to the respective non-irradiated control group are marked with an asterisk.

These UV-B radiation-modulated changes in the expressions of K 1, K 10 and involucrin were accompanied by a significant increase in the expression of c-Jun compared to control 6 and 24 hours after the irradiation (Figure 3). Moreover, we observed a significant increase in MMP-1 expression after 6 and 24 hours (Figure 3). In addition, a UV-B dose of 10 mJ/cm^2^ stimulated the release of IL-6 and IL-8 after 6 and 24 hours, with more profound effects 24 hours after irradiation (Figure 4).

## Discussion

The activation of a range of protective and reparative intracellular mechanisms and the production of various mediators stimulating the development of local inflammatory response are features of the complex stress response of keratinocytes to sublethal doses of UV radiation. In this study, we showed that a dose of UV radiation which did not induce complete cell death in human keratinocytes *in vitro* modulated the gene expressions of K 1, K 10 and involucrin. These effects were part of a complex reaction to UV radiation, which also included increased expression of c-Jun and MMP-1, and the production of IL-6 and IL-8.

To evaluate the mechanisms of keratinocyte response to UV radiation, a single dose of 10 mJ/cm^2^ of UV-B radiation (300±5 nm) was selected as the smallest dose with detectable biological effects. This dose did not induce complete death of keratinocytes under the conditions applied in our study, thus providing enough surviving cells for further analyses of the modulation of cell physiology. Similar keratinocyte sensitivity to UV-B radiation was also observed by other authors (Hunt *et al.*, 2006; Ishida & Sakaguchi, 2007).

The expressions of selected keratins and involucrin are recognized as markers of keratinocyte differentiation and maturation. To this date, inconsistent information exists about the modulation of keratin and involucrin expression by human keratinocytes in response to UV radiation. This study showed that the expression of K 1 and K 10 were increased after UV-B irradiation, suggesting that this acute response of keratinocytes to UV-B radiation is followed by induction of a higher level of keratinocyte differentiation status in the case of filament formation. Currently, the results for keratin expressions after UV-B irradiation of the skin are inconsistent, since one study found an increase in K 1 and K 10 in suprabasal located keratinocytes (Smith & Rees, 1994), some studies reported decreased K 1 and K 10 expression in keratinocytes (Bernerd & Asselineau, 1997; Horio *et al.*, 1993), and one study reported that UV-B radiation had no effect on K 1 gene expression (Lee *et al.*, 2002). The increase in K 10 could have an impact on the regulation of HaCaT response to UV radiation, particularly a decrease in cell proliferation, since K10 was shown to contribute to the inhibition of cell cycle entry (Paramio *et al.*, 1999). Accordingly, a loss of K 10 was reported to be connected with increased proliferation of keratinocytes (Reichelt *et al.*, 2004). K 1 has been suggested to participate in an inflammatory network in keratinocytes, since the absence of K 1 caused an increase in keratinocyte-autonomous IL-18 expression and release (Roth *et al.*, 2012).

In this study, involucrin expression was decreased but at the time of the most robust decrease in keratin expression, *i.e.*24 hours after irradiation, involucrin was unchanged. These results indicate distinct responses to UV irradiation of proteins involved in keratinocyte differentiation. In general, involucrin expression is known to be increased after UV irradiation and/or in sun-exposed skin (Bertrand-Vallery *et al.*, 2010; Bosset *et al.*, 2003; Gambichler *et al.*, 2008; Kwon *et al.*, 2008; Lee *et al.*, 2002), but there are also studies reporting decreased involucrin expression (Mammone *et al.*, 2000) or no effect of UV irrradiation on involucrin (Bernerd & Asselineau, 1997). These significant differences between the results obtained depend on the UVB source, the dose, and the biological model. It was also reported that other factors which produced a response to UV radiation, such as IL-1, TNF-α and c-JUN, were involved in involucrin induction in keratinocytes (Han *et al.*, 2012; Yano *et al.*, 2008), a finding that we did not observe.

The acute reaction of keratinocytes to UV radiation has been suggested to be mediated by early response transcription factor c-Jun, which is a part of the AP-1 complex stimulating inflammatory and other processes. In agreement with this, we observed increased gene expression of this transcriptional factor. It has been shown that c-Jun transcription factors participate in the UV-B-induced breakdown of the dermal extracellular matrix by inducing the expressions of a series of MMP responsible for collagen degradation (Fisher *et al.*, 1999; Soriani *et al.*, 2000). This reaction further continues with the activation of the MMP-1 gene, a direct downstream target of the AP-1 factor. In the present study, we observed increased MMP-1 expression 6 and 24 hours after single UV-B irradiation of keratinocytes. Increased MMP-1 expression after solar-simulated UV irradiation was reported in human skin *in vivo*, suggesting the importance of these *in vitro* observations (Lahmann *et al.*, 2001).

The response of keratinocytes to UV radiation comprises a strong positive feedback loop, which is responsible for the amplification of the reaction and leads to the undesirable manifestations of acute UV damage to the skin. In the present work, a UV-B-induced production of IL-6 and IL-8 was observed, which was in accordance with other authors presenting findings on various pro-inflammatory cytokines (Schwarz & Luger, 1989; Takashima & Bergstresser, 1996), including IL-1,IL-6 (Kirnbauer *et al.*, 1989) and IL-8 (Kondo *et al.*, 1993; Pernet *et al.*, 1999). The secretion of these cytokines may augment local immunological and inflammatory reactions following UV irradiation (Schwarz & Luger, 1989).

In conclusion, increases in the expression of the keratins K 1 and K 10 and a decrease in the expression of involucrin were described in UV-B-irradiated human keratinocytes. These changes are presented in the context of the typical keratinocyte response to UV-B radiation, which includes an increase in the expression of the stress-related transcriptional factor c-Jun and a consequent increase in MMP-1 expression, as well as the production of pro-inflammatory cytokines IL-6 and IL-8. All these changes were time dependent and more evident 24 hours after irradiation, when cell viability significantly decreased. On balance, these responses could be interrelated. Keratins may affect the ability of cells to proliferate or affect interleukin networks (Paramio *et al.*, 2001; Roth *et al.*, 2012). Conversely, inflammation response, *e.g.* the production of IL-1, was found to have a strong effect on the creation of hyperkeratosis, which included changes in keratin and involucrin levels (O'Shaughnessy *et al.*, 2010). Transcription factor AP-1 and its component c-Jun are induced by various stimuli, *e.g.* cytokines or stress stimuli, which in turn lead to the regulation of cell proliferation and differentiation (Shaulian & Karin, 2002).
